# Kinugasa Reactions in Water: From Green Chemistry to Bioorthogonal Labelling

**DOI:** 10.3390/molecules20046959

**Published:** 2015-04-16

**Authors:** Mariya Chigrinova, Douglas A. MacKenzie, Allison R. Sherratt, Lawrence L. W. Cheung, John Paul Pezacki

**Affiliations:** 1 Life Sciences Division, National Research Council of Canada, Ottawa, ON K1A 0R6, Canada; 2 Department of Chemistry, University of Ottawa, Ottawa, ON K1N 6N5, Canada

**Keywords:** nitrones, aqueous Kinugasa reaction, β-lactams, bioorthogonal, metabolic labelling

## Abstract

The Kinugasa reaction has become an efficient method for the direct synthesis of β-lactams from substituted nitrones and copper(I) acetylides. In recent years, the reaction scope has been expanded to include the use of water as the solvent, and with micelle-promoted [3+2] cycloadditions followed by rearrangement furnishing high yields of β-lactams. The high yields of stable products under aqueous conditions render the modified Kinugasa reaction amenable to metabolic labelling and bioorthogonal applications. Herein, the development of methods for use of the Kinugasa reaction in aqueous media is reviewed, with emphasis on its potential use as a bioorthogonal coupling strategy.

## 1. Introduction

Nitrones are emerging as biocompatible 1,3-dipoles that are tunable, hydrolytically stable, and highly reactive in cycloadditions with strained alkynes forming stable isoxazoline ring structures [1,2,3,4,5,6]. In addition to isoxazoline synthesis, nitrones are key precursors to diverse β-lactam structures through the copper(I)-catalyzed Kinugasa reaction with acetylides [7,8]. Since the initial report of the reaction between copper(I) acetylides and nitrones in pyridine in 1972 [8], the scope and limitations of the Kinugasa reaction have been explored by a number of research groups [9,10,11]. Recent progress in expanding the scope of the reaction has afforded the transition from requiring polar aprotic reaction media to an ‘on water’ approach, as well as employing detergents to allow for a micelle-promoted reaction to occur in aqueous medium [12]. The generation of aqueous-compatible Kinugasa variants as well as examination of the atomic connectivity of each product formed broadens the applicability as an efficient and modular coupling reaction.

In addition to the applications of the Kinugasa reaction for the synthesis of β-lactams, their usage in the context of bioorthogonal labelling is emerging [13]. Bioorthogonal labelling strategies, which are employed to chemically link reporter molecules to biomolecular targets in live environments, require the use of rapid and selective coupling reactions that proceed in a non-toxic manner under physiological conditions [14,15,16,17,18,19]. A wide variety of ligation strategies has been developed over the past decade for this purpose which makes use of exogenous functional groups that cause minimal disruption of the native biochemical processes occurring in the delicate reaction medium. These coupling reactions are propelled by a selection of driving forces, such as the release of ring strain [1,20,21], the expulsion of environmentally benign gasses [22,23], or the formation of strong amide bonds at the expense of weaker linkages executed through condensation [24] or rearrangement [13]. Despite the variety of reaction modes, each bioorthogonal labelling strategy is rooted by a common goal: to covalently link two modular components as efficiently as possible [25]. With this perspective in mind, existing chemistries can be rejuvenated and optimized for application in a variety of new environments. This short review highlights the strategies employed to transition the Kinugasa reaction to aqueous conditions, as well as the development of a particular Kinugasa variant—Copper(I) catalyzed Alkyne-Nitrone Cycloaddition with Rearrangement (CuANCR) [13]—as a bioorthogonal strategy for metabolic labelling.

## 2. Factors Affecting Catalysis in the Kinugasa Reaction 

Historically the Kinugasa reaction involves reacting copper acetylide with a nitrone in the presence of pyridine to form β-lactams [8]. The first step in the Kinugasa reaction mechanism is believed to proceed by cycloaddition of the organocuprate with the nitrone [7]. An alternative reaction that diverges away from β-lactam production can occur whereby homoalkyne dimerization products are formed through a Glaser type process. Oxygen-free conditions are often employed to prevent the Glaser reaction from occurring. As such copper(I) salts are typically used in the reaction or alternatively copper(II) salts can be used in the presence of a reducing agent, such as sodium ascorbate, to furnish the active copper(I) species [26]. However, it has been shown that copper(II) salts can still furnish β-lactam products in the absence of sodium ascorbate or other reducing species. It is believed that copper(II) can be reduced to the active copper(I) species by a Glaser reaction; this is further supported by isolation of the homo alkyne coupled product as well as X-Ray studies [27,28].

**Scheme 1:**
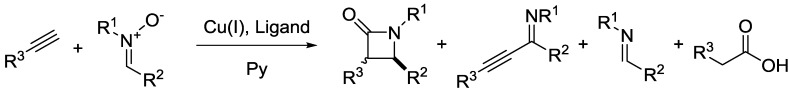
Products isolated from the Kinugasa reaction as observed by Muira *et al* [29].

Ligands and bases can have a significant effect on the product distribution of the Kinugasa reaction. Both phosphine and nitrogen based ligands can be employed, however, it has been shown that employing phosphine ligands, such as PPh_3_, PBu_3_, dppe, dppp, at elevated temperatures provides a diastereoisomeric mixture of β-lactams, 1-aza-1-buten-3-ynes, imines and carboxylic acid that are redox products (Scheme 1) [29].

β-Lactam products are isolated in low yields (6%–36%) when phosphine ligands are used, with the major products being the azaenyne and the redox products. Product selectivity is largely determined by the reactivity of the copper acetylide intermediate. 

**Scheme 2:**
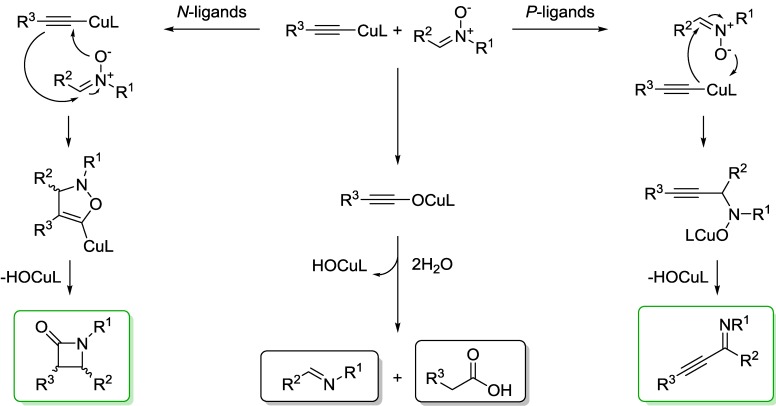
Reaction mechanism for the formation of different products isolated from the Kinugasa reaction employing phosphine and nitrogen-based ligands. Both the β-lactam and azaenyne products maintain the modular connectivity of the alkyne and nitrone appendages, while the imine and acid products are non-productive from a metabolic labelling standpoint.

Azaenyne products are most easily produced with phosphine ligands. It has been suggested that bidentate ligands bind tightly to the copper center which in turn increases the selectivity for the azaenyne product and suppresses β-lactam production. This is further supported by the fact that β-lactam products are only isolated at elevated reaction temperatures. It is possible that the phosphine ligand increases the electron density at the terminal alkyne making it less reactive towards a nucleophilic addition by the nitrone and more susceptible to nucleophilic attack on the nitrone (Scheme 2). Nitrogen ligands (e.g., bpy, phen, py), in contrast to phosphine ligands, do not require elevated temperatures for β-lactam synthesis; in fact, lower temperatures enhance production of β-lactams, while increased temperatures produce greater quantities of the imine and carboxylic acid redox products. It is believed that nitrogen ligands stabilize the monomeric copper acetylide making it more susceptible to cycloaddition with a nitrone (Scheme 2). A possible mechanism for the formation of the redox products begins with the oxygen atom of the nitrone coordinating to the copper of acetylide, followed by insertion into the C-Cu bond which furnishes the imine and an alkynyloxy copper species that is hydrolyzed to give the acid [29]. In addition to the effects of ligands on the Kinugasa reaction, nitrogen Brønsted bases are known to affect the diastereoselectivity of the reaction. In general, nitrogen bases with bulky substituents produce the syn diastereoisomer over the *anti* diastereoisomer. It has been suggested that the amine base might coordinate to the copper center, in addition to the ligand, to induce production of the *syn* diastereoisomer [30].

### 2.1. Water as the Solvent

**Scheme 3:**
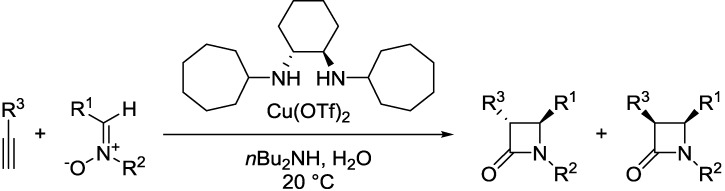
Enantioselective β-lactam synthesis under aqueous conditions.

Traditionally, Kinugasa reactions are performed in organic solvents, such as DMF, MeCN, and sometimes in neat pyridine. Basak *et al.* reported successful Kinugasa reaction with copper catalysis, in aqueous conditions in DMF/water, *t*-BuOH/water and MeCN/water [31]. The authors also performed the reaction in pure water, however, the efficiency and yield were reduced. Recently, the first asymmetric Kinugasa reaction has been performed “on” water [27]. Asymmetric induction was achieved using a C2-symmetric secondary diamine ligand to coordinate the copper species. Optimized conditions included using *n*Bu_2_NH as the base, copper(II) triflate as the copper source, water as the solvent, and a reaction temperature of 20 °C (Scheme 3).

*Reagents and Conditions*: Cu(OTf)_2_ (10 mol %), ligand (20 mol %), alkyne (0.2 mmol), nitrone (0.21 mmol), *n*Bu_2_NH (0.2 mmol), water (0.4 mL), 20 °C. Ar=4-EtO_2_CC_6_H_4_ [27].

The authors reported that the electronic character of the aromatic group attached to the nitrone carbon has no effect on the enantioselectivity of the reaction. Electron rich aromatic alkynes had a higher observed reactivity than electron poor aromatic alkynes. Olefin-conjugated alkynes had high enantiomeric excesses but lower diastereoselectivities. The electronic character of the aromatic substituent attached to the nitrogen of the nitrone has no effect on the enantiomeric excess; all β-lactams isolated had high enantiomeric excess. However, electron rich aromatic rings attached to the nitrogen produced β-lactams with much lower levels of diastereoselectivity compared to the electron poor aromatic ring counterparts. Decent to good results were obtained with heterocyclic and aliphatic side chains. 

Further studies were conducted on the reaction route and it was discovered that the reaction time has an effect on the β-lactam *syn/anti* ratio. With increasing reaction time the *anti/syn* β-lactam ratio increases, which indicates that as the reaction progresses the product epimerizes from the *syn* epimer to the *anti* epimer through a Brönsted acid/base sequence.

### 2.2. Micelle-Promoted Kinugasa Reaction

**Scheme 4:**
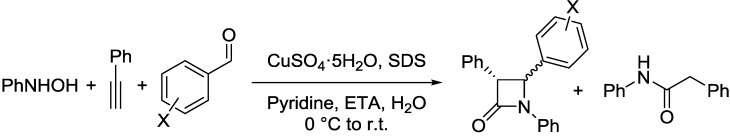
Synthesis of β-lactams by micelle-promoted Kinugasa reaction in water with the production of an amide side product not observed under anhydrous reaction conditions.

McKay *et al.* were the first to demonstrate the Kinugasa reaction in aqueous media promoted by surfactants [12]. Due to previous accounts of lower reactivity in water compared to aqueous organic solvent systems [31], micelles were employed to replace the organic solvent and help with solubility of the starting materials and the products, as well as promote the reaction by increasing the effective concentration of the reactants inside the micelles. The nitrone was synthesized *in situ* via the condensation of an aldehyde and a hydroxylamine in the presence of sodium dodecyl sulfate (SDS), and subsequently reacted with the alkyne to produce the desired β-lactams (Scheme 4). In addition to the β-lactam products, a newly observed amide by-product was also isolated. The amide was ruled out as a potential degradation product of the β-lactams by isolating the products and reintroducing them to the initial reaction conditions lacking the β-lactam precursors. No reaction giving rise to this by-product was observed in this case. This amide product is not a typical by-product of the Kinugasa reaction and its production may be unique to the employed aqueous conditions. 

With careful selection of the hydroxylamine employed, however, connectivity between the *N*-alkyl group of the resultant nitrone and the acetylene can be maintained after the modified Kinugasa reaction. Electron donating groups on the aldehyde resulted in lower combined β-lactams yield, while electron withdrawing groups generally showed lower production of the amide side product and significantly higher yield of the desired β-lactams. The desired products could be achieved in a yield of up to 85% depending on the optimized conditions used. Interestingly, living cellular systems, such as bacteria, for example, maintain elaborate and extensive networks of lipids and bio-polymers on the outer surface [32]. Depending on the composition and structure, such networks can simulate the surfactant activity by creating micelle-like environment for successful promotion of both the solubility of the starting materials, which suggests that the micelles used by McKay *et al.* may be biomimetic [12]. The latter led to the hypothesis that the Kinugasa reaction may be amenable to the labelling of living cells. 

## 3. Kinugasa Reaction for Bioorthogonal Chemistry

Bioorthogonal labelling involves the introduction of a biologically inert functional group into a biomolecule of interest *in vivo*, followed by specific ligation with a reporter (e.g., affinity or fluorescent tag) conjugated to a reactive partner [33]. Ideally, functional groups in bioorthogonal chemistry are nontoxic, cause minimal perturbation of their target biomolecules, and are selective for their reaction partners. Considering the vast array of functionalities in living systems, there is great interest for the development of new labelling strategies to add to the current bioorthogonal toolbox. From the organic chemistry perspective, bioorthogonal chemistry places one of the more rigorous sets of criteria on the labelling reactions compared to any other application. The main objective of these reactions is to produce the covalent connection between a label, usually incorporated biochemically or metabolically, and the tag. Therefore, to be considered for bioorthogonal labelling a reaction must be efficient, and must maintain the high yields of products or covalent connectivity of the starting materials, as well as the high reactivity in water or biological fluids as the solvent.

Recent developments allow the Kinugasa reaction to have the potential to be employed as bioorthogonal “click” chemistry reaction in biological labelling applications. Traditionally Kinugasa reaction effectiveness has been evaluated by the production of specific β-lactam products, their stereoselectivity and corresponding yields. These yields are commonly measured after product isolation and the yields of other products, from the initial reaction or due to β-lactam ring hydrolysis, are usually not even reported. Thus the perceived efficiency of the C-C bond production between the starting materials may appear low. However, the low stereoselectivity and even the poor desired product formation, from the synthetic point of view, are not necessarily impediments to a successful biological labelling strategy. From the bioorthogonal labelling perspective, the overall product yield of the Kinugasa reaction can be quiet high. If the newly formed C-C bond between the metabolic label and the tag stays intact, the labelling is successful.

In addition to high yield of probe-tag connectivity, bioorthogonal Kinugasa reagents must react fast in cell media and ideally cause minimal perturbation to the metabolic processes within cells. Thus there is a demand for optimization of the reaction conditions and reagents to produce a rapid reaction with minimal toxicity within a biological system. Similar to optimization of CuAAC, the copper source, the ligands and the base can be chosen based on their utility and lowered toxicity [17,18]. However, Kinugasa reaction outcome depends highly on the chosen components and reaction conditions, therefore the accommodation of toxicity may give rise to a possible decrease in reactivity. This is especially true for the choice of solvent. Organic solvents that are traditionally employed for Kinugasa reactions are generally toxic to cells [34,35,36]. Use of DMSO, DMF or MeCN can lead to increasing cell death, and is most likely to produce changes to cellular homeostasis and metabolism that will skew or misrepresent the results of the labelling experiments. Therefore, the development of the Kinugasa reaction under aqueous conditions is an exciting and new direction for this reaction and has recently been successfully applied in bioorthogonal labelling.

## 4. Bioorthogonal Labelling Applications

**Figure 1:**
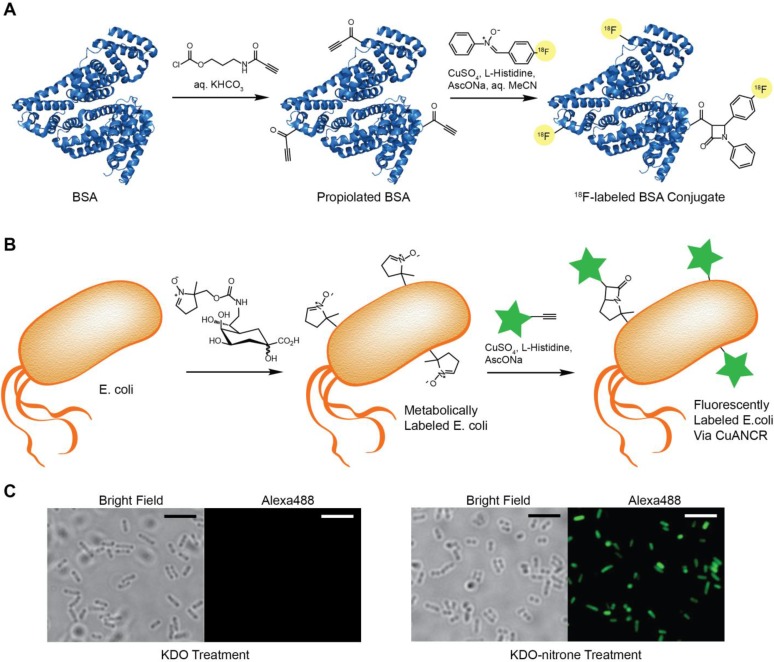
Bioorthogonal labelling by the Kinugasa reaction. (**A**) ^18^F-BSA conjugate formation by the radio-Kinugasa reaction by Cu(I)-histidine catalysis in aqueous acetonitrile. BSA (PDB 3V03) is propiolated using the indicated acylating agent, and then reacted with ^18^F-*N*-phenyl nitrone to produce an ^18^F-labelled BSA conjugate. Adapted from [37] (**B**) Metabolic labelling and fluorophore conjugation of living *E. coli* by copper-catalyzed alkyne-nitrone cycloaddition and rearrangement (CuANCR). Gram negative bacteria cultured in the presence of KDO-nitrone incorporate the functionalized sugar into the inner core of LPS molecules. Incorporated nitrone is detected after Cu(I)-histidine catalysis for the conjugation of an alkyne-tagged reporter. Adapted from [13] (**C**) Fluorescence microscopy of *E. coli* cultured in the presence of KDO (left) and KDO-nitrone (right) after CuANCR labelling with Alexa488-alkyne. Scale bar = 5 µm.

The bioorthogonal labelling potential of the Kinugasa reaction was initially demonstrated *in vitro* as a method to radio-label a protein sample [37]. Since there is an increasing demand for fluorine-18 labelled biomolecules for use as positron emission tomography (PET) tracers, there is an increasing demand for rapid and mild ^18^F incorporation methods as well. Zlatopolskiy *et al.* [37] built upon reports of the Kinugasa reaction under copper-catalyzed azide-alkyne cycloaddition (CuAAC) conditions [31], and applied a low-toxicity CuAAC method specifically developed for live-cell applications [17] to label a model protein, bovine serum albumin (BSA), with ^18^F. As shown in Figure 1, BSA was conjugated with the acylating agent, 3-propiolamidopropyl chloroformate, then ^18^F-labelled in aqueous MeCN via the Kinugasa reaction using Cu(I)-histidine catalysis.

Rapid kinetics and mild conditions highlight this Kinugasa reaction as a useful method for radio-labelling proteins and other biomolecules *in vitro* for PET tracer synthesis. Beyond protein labelling, this is the first description of ^18^F-labelled β-lactam formation, and has potential for applications in bacterial infection imaging [37].

As the Kinugasa reaction was being developed for rapid biomolecule radio-labelling and PET tracer synthesis, our group was uncovering *in vivo* applications of the Kinugasa reaction for the bioorthogonal labelling of living cells [13]. Recently, we and others [4,6,21,38] have demonstrated fast bioorthogonal reaction kinetics with nitrones by strain-promoted alkyne-nitrone cycloaddition (SPANC). Due to the stability of endocyclic nitrones and development of the Kinugasa reaction under aqueous conditions, we applied low-toxicity Cu(I)-histidine catalysis for β-lactam formation on living cell surfaces by copper-catalyzed alkyne-nitrone cycloaddition and rearrangement (CuANCR) [13]. This was initially demonstrated *in vivo* with mammalian cells, where sialylated glycans of human hepatoma (Huh-7) cells were labelled with mannosamine-alkyne, and detected by CuANCR with biotin-nitrone and FITC-streptavidin staining. Additionally, metabolic incorporation of the nitrone group into bacterial lipopolysaccharides was demonstrated when *E. coli* were cultured with nitrone-tagged 3-deoxy-D-manno-octulosonic acid (KDO), and detected by CuANCR with Alexa488-alkyne (Figure 1 B,C). This is the first example of metabolic incorporation of a nitrone group, and represents a new Cu(I)-catalyzed labelling reaction complimentary to CuAAC, that can selectively occur among functional groups present on cell surfaces. 

With cell surface labelling demonstrated for both eukaryotic and prokaryotic cell types, it is anticipated that CuANCR will be a valuable labelling strategy for biomolecules where either terminal alkyne or nitrone can be incorporated. Alkyne and nitrone tagged KDO were both incorporated by *E. coli* and detected via CuANCR [13]. Since KDO is an essential component of lipopolysaccharides, with metabolic labelling previously demonstrated for multiple species of Gram negative bacteria by CuAAC [39], it is expected that CuANCR can be applied interchangeably for labelling other bacteria as well. Our group is currently focused on further development of CuANCR as a bioorthogonal labelling method given its applications in the study of both mammalian and bacterial cells.

## 5. Conclusions/Summary 

In recent years the Kinugasa reaction has been successfully performed ‘on water’ and in aqueous media with the assistance of micellar catalysis. These advances in reaction scope have led to more green chemistry methods that allow for the large scale production of β-lactams in an economical manner without the need for organic solvents. Moreover, these advances have led to the adaptation of the Kinugasa reaction for bioorthogonal labelling of cells incorporating either alkyne or nitrone moieties in their surfaces. This opens the door to many applications whereby the Kinugasa reaction is used to functionalize living systems for the study of biomolecular processes.
