# Correction: Chigrinova, M., *et al.* Kinugasa Reactions in Water: From Green Chemistry to Bioorthogonal Labelling. *Molecules* 2015, *20*, 6959-6969

**DOI:** 10.3390/molecules20058303

**Published:** 2015-05-07

**Authors:** Mariya Chigrinova, Douglas A. MacKenzie, Allison R. Sherratt, Lawrence L. W. Cheung, John Paul Pezacki

**Affiliations:** 1 Life Sciences Division, National Research Council of Canada, Ottawa, ON K1A 0R6, Canada; 2 Department of Chemistry, University of Ottawa, Ottawa, ON K1N 6N5, Canada

The authors wish to make the following correction to this paper [1]: The author name “Paul Pezacki” should be “John Paul Pezacki”.

We apologize for any inconvenience caused to the readers. The online version will be updated, and the original will remain available on the article webpage.
